# Incidence of relapse of inflammatory protein‐losing enteropathy in dogs and associated risk factors

**DOI:** 10.1111/jvim.16561

**Published:** 2022-10-07

**Authors:** Jodie Green, Aarti Kathrani

**Affiliations:** ^1^ Royal Veterinary College Hatfield UK

**Keywords:** bowel, canine, lymphangiectasia, survival

## Abstract

**Background:**

Dogs with inflammatory protein‐losing enteropathy (iPLE) that attain remission may be at risk of subsequent relapse.

**Objectives:**

To determine the incidence of relapse of iPLE in dogs that have previously attained complete clinical and biochemical remission and identify associated risk factors.

**Animals:**

Seventy‐five client‐owned dogs diagnosed with iPLE.

**Methods:**

Medical records of dogs diagnosed with iPLE based on histopathology of intestinal biopsy specimens between March 2010 and March 2020 were retrospectively reviewed. Variables were recorded from the time of investigation at histopathologic diagnosis and subsequent follow‐up information was obtained from the records of referring veterinarians.

**Results:**

Twenty‐three dogs (31%) achieved sustained remission without documentation of relapse for at least 2 years. Nineteen dogs (25%) achieved remission, but then subsequently relapsed within 2 years of histopathologic diagnosis, and 33 dogs (44%) never achieved remission with disease‐associated death occurring a median of 19 (range, 3‐114) days after histopathologic diagnosis. Dogs that achieved remission and subsequently relapsed had significantly higher poor dietary compliance, as defined by frequent scavenging or changing from the recommended diet compared to dogs with sustained remission (*P* = .01).

**Conclusions:**

Inflammatory PLE is associated with a high rate of relapse in dogs. Ensuring owners adhere to dietary recommendations might help prevent subsequent relapse in dogs with iPLE that attain initial remission.

**Abbreviations**
CCECAI: canine chronic enteropathy clinical activity index
CIE: chronic inflammatory enteropathy
cPLI: canine pancreatic lipase immunoreactivity
CRP: C reactive protein
cTLI: canine trypsin‐like immunoreactivity
GI: gastrointestinal
IBD: inflammatory bowel disease
iPLE: inflammatory protein‐losing enteropathy
LPE: lymphoplasmacytic enteritis
NR: no remission
RR: remission‐relapsing
SPSS: Statistical Product and Service Solutions
SR: sustained remission
WSAVA: World Small Animal Veterinary Association

## 1 INTRODUCTION

Inflammatory protein‐losing enteropathy (iPLE) in dogs caused by chronic inflammatory enteropathy (CIE) is associated with a guarded prognosis, and disease‐associated death occurs in approximately 50% of cases.^1^ Several studies therefore have focused on prognostic indicators of outcome, where outcome is defined as survival versus nonsurvival for a determined follow‐up period.^1^, ^2^, ^3^, ^4^, ^5^, ^6^, ^7^, ^8^, ^9^ Factors identified as predictors of negative outcome vary among studies and have included vomiting, monocytosis; abnormal blood urea nitrogen concentration, high C‐reactive protein (CRP) concentration, low serum albumin concentration; hypovitaminosis D, body weight, and canine chronic enteropathy clinical activity index (CCECAI).^1^, ^2^, ^3^, ^4^, ^5^, ^6^, ^7^, ^8^, ^9^

Studies with long‐term follow‐up to assess outcome, defined as >6 months, in dogs with iPLE are currently lacking. Furthermore, few studies have documented outcome with regard to resolution of both clinical signs and hypoalbuminemia.^2^, ^5^, ^10^ One study assessing Yorkshire terriers with iPLE found that long‐term follow‐up was achieved in 23 dogs.^5^ Thirteen of 23 dogs (57%) had complete resolution of clinical signs and normalization of serum albumin concentration and 3 achieved partial resolution of signs, albumin concentration or both with a median survival time of 44 months.^5^ Four of the dogs that achieved complete remission however subsequently relapsed with or without hypoalbuminemia 3 to 20 months after diagnosis, resulting in disease‐associated death. Factors associated with clinical relapse in these dogs were not investigated.

To our knowledge, no other study has ascertained from the surviving population of dogs with iPLE what proportion of dogs that achieve clinical and biochemical remission remain in remission versus those that relapse. The incidence of and risk factors for relapse in dogs diagnosed with iPLE are therefore currently unknown. Understanding the incidence of relapse and factors associated with relapse in dogs with iPLE could positively affect earlier or ongoing monitoring and treatment strategies and could impact patient outcome.

Our aims were, first, to ascertain what proportion of dogs that achieved remission remained in remission versus those that went on to relapse. Second, we aimed to identify any associated risk factors from the time of histopathologic diagnosis that were predictive of subsequent relapse in dogs that attained remission. We defined remission as resolution of both clinical signs and hypoalbuminemia and relapse as recurrence of both clinical signs and hypoalbuminemia after initial remission.

## 2 MATERIALS AND METHODS

### 2.1 Study animals

The medical records at the Queen Mother Hospital for Animals, Royal Veterinary College in the United Kingdom were retrospectively searched between March 2010 and March 2020 for dogs diagnosed with an iPLE. Inclusion criteria consisted of clinical signs consistent with PLE, hypoalbuminemia (<26.3 g/L) at presentation, a CIE with or without lymphangiectasia diagnosed on histopathology of endoscopically‐obtained intestinal biopsy specimens and adequate further diagnostic testing to exclude other causes of hypoalbuminemia and gastrointestinal signs. A minimum follow‐up period of 2 years was required for all dogs included in the study, unless they were euthanized or died as a result of their iPLE. All follow‐up data was acquired from subsequent visits or from the records of referring veterinarians. Dogs were excluded from the study if a histopathologic diagnosis was not obtained from intestinal biopsy, if a neoplastic cause was identified on histopathologic examination or if initial serum biochemistry, abdominal imaging and urinalysis results failed to exclude a hepatic or renal cause for the hypoalbuminemia. Dogs also were excluded from the study if they had incomplete medical records or inadequate follow‐up data. In total, 75 dogs met the inclusion criteria.

### 2.2 Diagnostic evaluation

For all 75 dogs, diagnostic evaluation included CBC, serum biochemistry, urinalysis, complete abdominal ultrasound examination by a board‐certified veterinary radiologist and upper gastrointestinal endoscopy with collection of biopsy specimens. All histopathologic diagnoses were assigned by a board‐certified veterinary pathologist. Further diagnostic testing included serum cobalamin concentration in 66 dogs (88%), basal cortisol concentration or ACTH stimulation testing in 50 dogs (67%), quantitative canine pancreatic lipase immunoreactivity (CPLi) in 27 dogs (36%), serum trypsin‐like immunoreactivity (cTLI) in 14 dogs (19%), pre‐ or pre‐ and postprandial bile acid concentrations in 6 dogs (8%), fecal parasitology using zinc sulfate flotation with centrifugation in 62 dogs (83%), empirical treatment with fenbendazole in 23 dogs (30%), urine protein: creatinine ratio in 25 dogs (33%), and lower gastrointestinal endoscopy and biopsies in 40 dogs (53%).

### 2.3 Outcome classification

The medical records were assessed and the following outcomes were defined for each of the 75 dogs included in the study:

#### 2.3.1 Remission

Dogs were defined as entering remission if they achieved both resolution of clinical signs and documented resolution of hypoalbuminemia (>26.3 g/L).

#### 2.3.2 Sustained remission

Dogs were categorized as being in sustained remission (SR) if they achieved and remained in clinical and biochemical remission for 2 years after histopathologic diagnosis.

#### 2.3.3 Remission‐relapsing

Dogs were categorized as remission‐relapsing (RR) if, having initially achieved clinical and biochemical remission, they had recurrence of both clinical signs and hypoalbuminemia (<26.3 g/L) within 2 years of histopathologic diagnosis.

#### 2.3.4 No remission

Dogs were categorized as no remission (NR) if they never achieved resolution of clinical signs and hypoalbuminemia and therefore were euthanized or died as a result of their disease.

### 2.4 Data collection and assessment of risk factors

The following information was collected and evaluated as risk factors for all dogs in SR, RR, and NR categories at the time of histopathologic diagnosis:

#### 2.4.1 Clinical history and presenting signs

Breed, sex, and neuter status were recorded and assessed as categorical variables. Presenting signs were assessed and categorized as diarrhea and weight loss (1), diarrhea weight loss and reduced appetite (2), diarrhea, weight loss, decreased appetite and vomiting (3), single or bicavitary effusions with or without peripheral edema in the absence of gastrointestinal signs (4), single or bicavitary effusions with or without peripheral edema in the presence of gastrointestinal signs (5). Diet before presentation was assessed and categorized as hydrolyzed, low fat (<20% fat on a metabolizable energy basis), limited‐ingredient novel protein diet or other. Dogs were assessed and categorized into 3 groups based on their year of histopathologic diagnosis: 2010 to 2013, 2014 to 2016, and 2017 to 2020. Treatment before histopathologic diagnosis was assessed and categorized as no treatment, immunosuppressive treatment, antibiotics or antibiotics and immunosuppressive treatment. Age (years), body weight (kg), and duration of presenting signs (days) before histopathologic diagnosis were recorded and assessed as continuous variables.

#### 2.4.2 Laboratory and histopathologic findings

Serum total protein, serum albumin, serum globulin, and serum cholesterol concentrations were recorded at the time of histopathologic diagnosis and assessed as continuous variables. Serum cobalamin concentration recorded within 1 month of histopathologic diagnosis was available and assessed for 66 dogs (88%). Histopathologic diagnosis was assessed and categorized as lymphoplasmacytic enteritis (LPE) (1), LPE and neutrophilic enteritis (2), LPE with eosinophilic enteritis (3), or CIE with lacteal dilatation (4). The World Small Animal Veterinary Association (WSAVA) gastrointestinal standardization grading scheme scores for both stomach and duodenum were available and assessed as ordinal variables for 42 dogs (56%).^11^

#### 2.4.3 Treatment after histopathologic diagnosis

Treatment with prednisolone was assessed and categorized as received or not received. Commencement of a second immunosuppressive agent was assessed and categorized as cyclosporine (1), azathioprine (2), chlorambucil (3), or no second agent (4). Type of dietary treatment also was assessed and categorized as hydrolyzed, limited‐ingredient novel protein or low‐fat diet. Dose of prednisolone treatment (mg/kg), time from histopathologic diagnosis to commencement of prednisolone treatment (days) and time from histopathologic diagnosis to starting a second immunosuppressive agent (days) were recorded and assessed as continuous variables.

The following information also was recorded and evaluated as risk factors for dogs in the SR and RR categories:

#### 2.4.4 Remission

For dogs in the SR and RR groups, the following information was recorded and assessed as continuous variables: time from histopathologic diagnosis to documented remission (days), total protein and albumin concentrations at remission, time from histopathologic diagnosis to prednisolone dose of 0.5 mg/kg (days), duration of prednisolone treatment (days), and survival time from histopathologic diagnosis (days). Information on dietary compliance was obtained from clinical notes and was assessed and categorized as poor, good or unknown. Dietary compliance was defined as poor if dogs had been changed to a nonrecommended diet type, were noted to be receiving additional food or scavenging. Dietary compliance was defined as good if dogs were still receiving the recommended diet type (hydrolyzed, low fat, or limited‐ingredient novel protein) with no record of scavenging at the time of clinical relapse in the RR group or by 2 years after histopathologic diagnosis in the SR group.

#### 2.4.5 Remission‐relapsing

For dogs in the RR group, the following information also was recorded: time from histopathologic diagnosis to relapse (days), time from documented clinical and biochemical remission to relapse (days), serum albumin concentration at relapse, achievement of second remission (categorized as yes or no), and time from documented relapse to second remission (days).

### 2.5 Statistical analysis

To evaluate risk factors, data collection, checking, and cleaning were performed in Microsoft Excel (2021). Categorical data were summarized by count and percentage. Median and range were calculated for continuous variables. The data were imported into IBM SPSS (Statistical Product and Service Solutions) version 28 statistical software for analysis. Statistical analyses were carried out to identify variables that were significantly different among the NR, SR, and RR groups. For continuous data, a Shapiro‐Wilks test was used to assess normality. For normally‐distributed data, 1‐way analysis of variance (ANOVA) was used to compare data among NR, SR, and RR groups and an independent *t*‐test was used to compare data between SR and RR groups only. For nonnormally distributed data, a Kruskal‐Wallis test was used to compare data among NR, SR and RR groups and Mann‐Whitney *U* test was used to compare data between the SR and RR groups. A chi‐squared test was used to compare categorical data among the NR, SR, and RR groups. Significance was defined as *P* < .05 for all analyses and Tukey's post hoc analysis was used for all significant findings.

## 3 RESULTS

### 3.1 Descriptive statistics

#### 3.1.1 Study animals

Seventy‐five dogs diagnosed with iPLE met the inclusion criteria: 12 male intact dogs, 30 male neutered dogs, 4 female intact dogs and 29 female neutered dogs. The median (range) age at histopathologic diagnosis was 6 (1–15) years and the median (range) body weight was 15.2 (4.1‐67.7) kg. There were 32 different breeds included: Staffordshire Bull terrier (12), cross‐breed (6), Cavalier King Charles spaniel (5), Pug (5), German shepherd (4) Miniature schnauzer (3), Bichon frise (3), Golden retriever (3), Dogue de Bordeux (2), Miniature dachshund (2), Yorkshire terrier (2), Labrador retriever (2), Jack Russell terrier (2), Rottweiler (2), Hungarian viszla (2), English bulldog (2), and 1 each of the following: Shiba Inu, Airedale terrier, Neapolitan mastiff, Cocker spaniel, Tibetan terrier, Leon Berger, Bull mastiff, Brussels Griffon, English Springer spaniel, Whippet, Weimaraner, Border collie, Shetland sheepdog, and English pointer.

#### 3.1.2 Clinical signs

Median (range) duration of clinical signs before histopathologic diagnosis was 31 (3‐855) days. Nineteen dogs (25%) had diarrhea and weight loss, 8 dogs (10%) had diarrhea weight loss and decreased appetite, 28 dogs (37%) had diarrhea, weight loss, decreased appetite and vomiting, 4 dogs (5%) had single or bicavitary effusions, peripheral edema, or both in the absence of gastrointestinal signs, 15 dogs (20%) had single or bicavitary effusions, peripheral edema or both in the presence of gastrointestinal signs.

#### 3.1.3 Laboratory and histopathologic findings

At the time of histopathologic diagnosis, median serum total protein concentration was 33.8 g/L (range, 18.4‐69.5; reference interval [RI], 54.9‐75.3 g/L), median serum albumin concentration was 16.7 g/L (range, 8.1‐25.5; RI, 26.3‐38.2 g/L), median serum globulin concentration was 16.5 g/L (range, 12‐40.7; RI, 23.4‐42.2 g/L), and median serum cholesterol concentration was 2.6 mmol/L (range, 1.2‐4.3; RI, 3.2‐6.2 mmol/L). Thirty‐four of 66 dogs (51%) for which serum cobalamin concentration was recorded had concentrations below the limit of the RI of 150 ng/L. On duodenal histopathologic diagnosis: 49 dogs (65%) were diagnosed with CIE and 26 dogs (35%) were diagnosed with CIE and lacteal dilatation. Of the 75 dogs, 42 dogs (56%) had lymphoplasmacytic enteritis, 19 dogs (25%) had lymphoplasmacytic and neutrophilic enteritis, 9 dogs (12%) had lymphoplasmacytic and eosinophilic enteritis and 5 dogs (7%) had lymphoplasmacytic, neutrophilic and eosinophilic enteritis.

#### 3.1.4 Treatment after histopathologic diagnosis

Fifty‐one dogs (68%) received a hydrolyzed diet, 14 dogs (19%) received a limited‐ingredient novel protein diet, 5 dogs (7%) received a gastrointestinal low‐fat diet, and 5 dogs (7%) were euthanized before initiating dietary treatment. Thirteen dogs (17%) received solely dietary treatment and 56 dogs (79%) received prednisolone treatment at a median (range) starting dosage of 2 (0.7‐5) mg/kg. Median (range) time from histopathologic diagnosis to starting prednisolone treatment was 0 (0‐119) days. Thirty‐three dogs (44%) did not receive a second immunosuppressive agent, 28 dogs (37%) received cyclosporine, 8 dogs (10%) received chlorambucil and 4 dogs (5%) received azathioprine. Median (range) time from histopathologic diagnosis to starting a second immunosuppressive agent was 5 (0‐210) days.

#### 3.1.5 Outcome

##### No remission

Thirty‐three dogs (44%) never achieved clinical and biochemical remission and either died or were euthanized in a median (range) of 19 (3‐114) days after histopathologic diagnosis.

##### Remission

Forty‐two dogs (56%) achieved clinical remission with resolution of clinical signs and hypoalbuminemia at a median (range) of 50 (10‐660) days. Median (range) serum albumin concentration at remission was 29.0 (26.3‐34.3) g/L. Seven dogs (16%) that achieved remission received dietary treatment alone and 39 dogs (86%) received dietary and immunosuppressive treatment. Of the 7 dogs that achieved remission with dietary treatment alone, 3 had lacteal dilatation on histopathology.

##### Sustained remission

Twenty‐three dogs (31%) achieved SR for 2 years after diagnosis. Three‐year follow‐up data was available for 16 of the 23 dogs and 4 year follow‐up data was available for 14 of the 23 dogs with no evidence of clinical or biochemical relapse.

##### Remission‐relapsing

Nineteen dogs (25%) had a relapse of their condition within 2 years of histopathologic diagnosis. The median (range) time from histopathologic diagnosis to relapse was 209 (37‐730) days and the median (range) time from remission to relapse was 84 (14‐607) days. Median (range) serum albumin concentration at the time of relapse was 21.7 (14‐24.7) g/L.

Of the 19 dogs that relapsed, 10 dogs (52%) achieved a second remission within a median (range) of 45 (14‐91) days. Nine dogs (48%) were euthanized or died as a result of their clinical relapse. Four dogs (40%) that survived their second remission were documented to suffer from >1 subsequent relapse of their disease.

In total, 42 dogs (56%) died as a result of their iPLE or because of a relapse of their iPLE within 2 years of histopathologic diagnosis.

## 4 STATISTICAL ANALYSIS OF RISK FACTORS

### 4.1 NR, SR, and RR

Summary statistics for risk factors are shown in Table 1. No significant difference was found when comparing the following risk factors among the 3 groups, NR, SR, and RR:

**TABLE 1:** Summary of variables identified in 75 dogs diagnosed with inflammatory protein‐losing enteropathy

Risk factor		NR^a^ (n = 23)	RR^b^ (n = 19)	SR^c^ (n = 33)	*P* value
Age (years)	Median (range)	7.5 (2‐13)	8 (2‐11)	6 (1‐15)	.2
Body weight (kg)	Median (range)	16.9 (5.1‐45.4)	9.3 (5.8‐67.8)	16.6 (6‐48.9)	.39
Date of histopathologic diagnosis					.13
	2010‐2013	10	2	11	
	2014‐2016	12	5	7	
	2017‐2020	11	12	5	
Presenting signs^d^	(1)	10	3	6	.47
	(2)	2	3	3	
	(3)	11	8	9	
	(4)	2	3	0	
	(5)	8	2	5	
Duration of presenting signs (d)	Median (range)	44 (3‐855)	61 (6‐220)	24 (2‐184)	.09
Albumin concentration (g/L)	Median (range)	16.7 (8.1‐24.2)	16.2 (10.3‐25.5)	15.9 (11.7‐22.9)	.98
Globulin concentration (g/L)		16.5 (8.3‐17.5)	20 (14.5‐26.2)	16.2 (12.8‐40.7)	.07
TP concentration (g/L)^e^	Median (range)	32.5 (21.5‐50.1)	36.6 (26‐48)	32.4 (25‐59.5)	.16
Cholesterol concentration (mmol/L)	Median (range)	2.6 (1.2‐4.3)	2.97 (1.8‐3.9)	2.35(1.9‐4.3)	.2
Histopathologic diagnosis^f^	1 LP	10	9	3	.12
	2 LP, N	7	3	9	
	3 LP, E (±N)	2	2	3	
	4 Lacteal dilatation	14	3	9	
WSAVA score stomach	Median (range)	2 (0‐7)	1 (0‐6)	2 (0‐7)	.5
WSAVA score duodenum	Median (range)	7 (3‐10)	6 (1‐13)	7 (3‐10)	.5
Dietary treatment	1 Hydrolyzed	18	15	16	.23
	2 Novel	6	3	5	
	3 Low fat	3	1	2	
Prednisolone treatment	1 Yes	24	18	17	.13
	2 No	9	1	6	
Prednisolone dosage (mg/kg)	Median (range)	2 (1‐5)	2 (0.7‐3)	2.5 (1‐4)	.98
Time from diagnosis to starting prednisolone (d)	Median (range)	0 (0‐36)	0 (0‐119)	0 (0‐28)	.09
Second immunosuppressive agent	0 None	17	6	14	.82
	1 Cyclosporine	12	9	7	
	2 Chlorambucil	3	3	1	
	3 Azathioprine	1	1	1	
Time from diagnosis to second immunosuppressive agent (d)	Median (range)	2.5 (0‐210)	28 (0‐126)	0 (0‐60)	.08
Time from diagnosis to remission (d)	Median (range)	N/A	62 (28‐660)	38 (10‐330)	.2
Time from diagnosis to 0.5 mg/kg prednisolone (d)	Medium (range)	N/A	96 (28 to >730)	141 (28‐399)	.87
Albumin concentration at remission (g/L)	Median (range)	N/A	29 (26.2‐32.2)	289 (26.3‐35)	.73
Dietary compliance	0 Unknown	N/A	1	2	.01
	1 Good		0	15	
	2 Poor		18	6	

*Note*: Statistical analyses were used to identify variables that were significantly different between dogs that never achieved clinical and biochemical remission, dogs that achieved sustained remission and dogs that achieved remission and relapsed.

^a^ No remission.

^b^ Remission‐relapsing.

^c^ Sustained remission.

^d^ Presenting signs: (1) diarrhea and weight‐loss; (2) diarrhea weight‐loss and reduced appetite; (3) diarrhea, weight‐loss, reduced appetite, and vomiting; (4) single or bicavitary effusions with or without peripheral edema in the absence of gastrointestinal signs; and (5) single or bicavitary effusions with or without peripheral edema in the presence of gastrointestinal signs.

^e^ Total protein.

^f^ Histopathologic diagnosis: (1) lymphoplasmacytic enteritis; (2) lymphoplasmacytic and neutrophilic enteritis; (3) lymphoplasmacytic and eosinophilic enteritis; and (4) chronic inflammatory enteropathy with lacteal dilatation.

Breed (*P* = .49), sex and neuter status (*P* = .57), age (*P* = .2), body weight (*P* = .39), date of histopathologic diagnosis (*P* = .13), presenting signs (*P* = .47), duration of signs before histopathologic diagnosis (*P* = .09), histopathologic diagnosis (*P* = .12); treatment before histopathologic diagnosis (*P* = .55), serum albumin concentration (*P* = .98), total protein concentration (*P* = .16), serum globulin concentration (*P* = .07), serum cholesterol concentration (*P* = .2), serum cobalamin concentration (*P* = .57), WSAVA histopathologic scores stomach (*P* = .5), WSAVA histopathologic scores duodenum (*P* = .5), type of dietary treatment (*P* = .23), treatment with prednisolone (*P* = .13), starting dose of prednisolone (*P* = .98), days from diagnosis to commencement of prednisolone treatment (*P* = .09), commencement of second immunosuppressive agent (*P* = .82) and number of days from diagnosis to starting a second immunosuppressive agent (*P* = .08).

#### 4.1.1 SR and RR

No significant difference was found when comparing the following risk factors between the SR and RR groups:

Days from diagnosis to documented remission (*P* = .2), serum albumin concentration at remission (*P* = .73) and days until prednisolone dose of 0.5 mg/kg (*P* = .87).

A significant difference was found when comparing dietary compliance between the SR and RR groups (*P* = .01). Remission‐relapsing dogs had significantly higher poor dietary compliance than did SR dogs. In the RR group, the dietary compliance of 1 dog could not be reliably established and from the remaining 18 dogs, only 1 dog was receiving the prescribed diet type at the time of relapse. However, this dog was reported to be a scavenger. In the SR group, dietary compliance for 2 out of 23 dogs could not be reliably established, however 15 of 21 dogs (71%) were reported to have had good compliance and were still solely receiving their prescribed diet type at least 2 years after histopathologic diagnosis.

For dogs in the SR group, 3 had formula types changed within the same category of diets (hydrolyzed [n = 2] and limited‐ingredient novel protein [n = 1]) before attaining remission. For dogs in the RR group, 1 was transitioned from a hydrolyzed to a limited‐ingredient novel protein diet and 2 had changed formula types within the same category of diets (hydrolyzed [n = 2]) before attaining remission.

## 5 DISCUSSION

Long‐term studies to assess the incidence of relapse of iPLE in dogs that achieve initial clinical and biochemical remission are currently lacking.^1^, ^2^, ^3^, ^4^, ^5^, ^6^, ^7^, ^8^, ^9^, ^10^ Our study aimed to determine the incidence of relapse of iPLE in dogs that have previously attained complete clinical and biochemical remission and identify associated risk factors. In our study, almost half of the dogs that achieved initial biochemical and clinical remission of their iPLE suffered a relapse of their condition (RR) within 2 years of histopathologic diagnosis. In humans, periods of remission and relapse are characteristic of inflammatory bowel disease (IBD) and are often hard to predict.^12^ Factors associated with an increased risk of relapse of IBD in humans include age, sex, duration of clinical signs, increased CRP concentration, corticosteroid requirement at onset, fecal calprotectin, and diet.^12^ Identifying IBD patients with multiple risks factors for relapse promotes earlier and more aggressive interventions, more frequent monitoring and improves patient outcomes.^12^ Identifying risk factors associated with relapse of iPLE in dogs therefore may be beneficial for ongoing monitoring and management strategies.

The only risk factor found to be significantly different between SR and RR groups in our study was dietary compliance. Poor dietary compliance, defined as changing diets to a nonrecommended diet type, receiving additional food or scavenging, was significantly higher in the RR group compared to the SR group. Dietary management is the mainstay of treatment for dogs with iPLE, but the role that long‐term dietary modification plays in achieving SR is unknown.^1^, ^13^, ^14^, ^15^ In both dogs and humans with IBD, subclinical mucosal inflammation can persist despite clinical remission, which may contribute to a risk of relapse when the inflammatory process reaches a critical intensity.^6^, ^13^, ^16^, ^17^ For dogs with CIE, hydrolyzed and limited‐ingredient novel protein diets remove antigenic sources and decrease the enteric inflammatory response, which may help achieve SR.^13^, ^14^ For dogs with lymphangiectasia or lacteal dilatation, low‐fat diets can be very effective, as demonstrated in several studies.^10^, ^18^, ^19^ Therefore, possible explanations for the association between poor dietary compliance and relapse in our study are that dogs may become sensitized to ingredients in their food or suffer adverse food reactions causing enteric inflammation and triggering a relapse of their iPLE when the implicated ingredients are reintroduced.^20^ Additionally, because some of the dogs in our study had evidence of lacteal dilatation on histopathology, consuming higher amounts of fat also may have triggered a relapse. However, not all dogs with poor dietary compliance in our study had a relapse of clinical signs. The reason why these dogs did not suffer a relapse is unclear, although concurrent immunosuppressive medication may have adequately suppressed inflammation in some cases.^14^ Interestingly, a previous study found that dogs with steroid‐resistant protein losing‐enteropathy were able to attain remission after a change in diet.^15^ In our study unfortunately only a few dogs underwent changes in diet after diagnosis, and therefore the effects these changes had on our study outcome is unknown and should be investigated in future studies.

Because our study was retrospective, the association between poor dietary compliance and relapse also could have been influenced in part by bias. Owners of dogs that relapse are more likely to seek veterinary attention and undergo more thorough examination of current dietary management. In contrast, owners with dogs in remission are less likely to report episodes of scavenging or supplementary feeding if the dogs are clinically well.

In our study, the overall incidence of poor dietary compliance was high in both SR and RR groups, but the reasons for poor dietary compliance were not investigated. Dietary compliance may be a particular challenge for owners in the treatment of iPLE because of the potential lower palatability of hydrolyzed or low‐fat diets, which could become more noticeable as glucocorticoid doses are decreased after remission.^21^ Growing aversion to hydrolyzed diets also could be confused with hyporexia as an initial sign of relapse, causing owners to change diets.^22^, ^23^ Alternatively, owners may not be aware of the importance of long‐term dietary management. Prospective studies therefore are warranted to investigate the relationship between dietary challenge and relapse in dogs with iPLE and to establish the benefit of long‐term dietary management for SR.

Our study did not identify significant risk factors for relapse other than poor dietary compliance. However, the retrospective nature of our study prevented investigation of a number of potential risk factors, including CCECAI, CRP, serum 25(OH) vitamin D concentration and blood urea concentration, which all have been associated with poor prognosis.^6^, ^7^ Duration of clinical signs before diagnosis is a poor prognostic indicator for relapse in humans with IBD, but it was not found to be a significant risk factor for iPLE relapse in our study.^12^ One explanation is that the wide variety of dietary and steroid interventions by referring veterinarians before histopathologic diagnosis may have affected results. Additionally, people may not be as successful assessing the onset of clinical signs in their pets as compared to themselves. Time from diagnosis to biochemical remission previously has been associated with long‐term survival in dogs with iPLE,^5^ but time from diagnosis to remission was not associated with achieving SR in our study. This finding may be related to the difference in definition of long‐term survival among studies. However, because of the retrospective nature of our study, significant variability occurred in follow‐up data among referring veterinarians. Intervals for follow‐up laboratory tests were not standardized, which could have impacted recorded duration from histopathologic diagnosis to documented remission.

In our study, relapse of iPLE was associated with a poor prognosis, with 50% of dogs dying or being euthanized as a result of their disease process. Previous studies have reported even lower survival rates.^5^ Many dogs that survived their first relapse also went on to suffer additional episodes of relapse in the future, a pattern more characteristic of IBD in humans.^12^ The poor prognosis associated with relapse is likely the result of a number of factors including disease progression, unresponsiveness to treatment, and financial constraints. However, a limitation of our study was that relapse was defined as recurrence of clinical signs and hypoalbuminemia, but repeat confirmation of histopathologic diagnosis was not required. Only 1 dog in the RR group had repeat endoscopic biopsies and repeat histopathologic diagnosis of lymphoplasmacytic enteritis. Clinical relapse in some dogs therefore could have been the result of a concurrent condition or neoplasia, which may have negatively skewed prognosis. Furthermore, because ileal biopsies were not performed in all cases, intestinal neoplasia, lymphangiectasia or lacteal dilatation could have been missed in some cases.^3^ In humans with IBD, shorter times between remission and relapse are associated with poorer prognosis and more frequent relapses.^12^ This association was not seen in our study, which could have been a result of small sample size.

Other limitations of our study also occurred as a result of the retrospective study design, including involvement of several clinicians in the cases resulting in variable dosages of prednisolone, as well as diet choice associated with clinician preference, because there currently is no definitive consensus for the treatment of these cases. Not all dogs had repeat serum biochemistry performed if episodes of diarrhea occurred after remission, which could have led to an underestimation of the number of relapse cases. However, dogs reported to have had episodes of diarrhea without repeat biochemistry after remission only were included in our study (and defined as SR) if the episodes of diarrhea resolved without additional medication or changes to medication they were receiving at the time.

In conclusion, our study emphasizes the substantial proportion of dogs that achieve clinical remission of iPLE and suffer relapses of their condition. Dogs that relapsed were found to have worse dietary compliance than those that remained in remission. Therefore, ensuring owners adhere to dietary recommendations might help prevent subsequent relapse in dogs with iPLE that attain initial remission. Prospective studies are required to investigate the relationship between dietary compliance and relapse in dogs with iPLE and to identify additional risk factors. Identification of risk factors will help improve monitoring and treatment strategies to try to decrease the incidence of relapse of iPLE and improve patient outcome.

## CONFLICT OF INTEREST DECLARATION

Authors declare no conflict of interest.

## OFF‐LABEL ANTIMICROBIAL DECLARATION

Authors declare no off‐label use of antimicrobials.

## INSTITUTIONAL ANIMAL CARE AND USE COMMITTEE (IACUC) OR OTHER APPROVAL DECLARATION

The Royal Veterinary College granted ethical approval for the study (URN SR2020‐0255).

## HUMAN ETHICS APPROVAL DECLARATION

Authors declare human ethics approval was not needed for this study.

## References

[1] Craven M , Washabau R . Comparative pathophysiology and management of protein‐losing enteropathy. J Vet Intern Med. 2019;33:383‐402. 30762910 10.1111/jvim.15406 PMC6430879

[2] Craven M , Simpson J , Ridyard A , et al. Canine inflammatory bowel disease: retrospective analysis of diagnosis and outcome in 80 cases (1995‐2002). J Small Anim Pract. 2004;45:336‐342. 15266855 10.1111/j.1748-5827.2004.tb00245.x

[3] Nakashima K , Hiyoshi S , Ohno K , et al. Prognostic factors in dogs with protein‐losing enteropathy. Vet J. 2015;205:28‐32. 26025135 10.1016/j.tvjl.2015.05.001

[4] Salavati Schmitz S , Gow A , Bommer N , Morrison L , Mellanby R . Diagnostic features, treatment, and outcome of dogs with inflammatory protein‐losing enteropathy. J Vet Intern Med. 2019;33:2005‐2013. 31381203 10.1111/jvim.15571 PMC6766500

[5] Simmerson S , Armstrong P , Wünschmann A , et al. Clinical features, intestinal histopathology, and outcome in protein‐losing enteropathy in Yorkshire Terrier dogs. J Vet Intern Med. 2014;28:331‐337. 24467282 10.1111/jvim.12291 PMC4857982

[6] Allenspach K , Wieland B , Gröne A , Gaschen F . Chronic enteropathies in dogs: evaluation of risk factors for negative outcome. J Vet Intern Med. 2007;21:700‐708. 17708389 10.1892/0891-6640(2007)21[700:ceideo]2.0.co;2

[7] Equilino M , Théodoloz V , Gorgas D , et al. Evaluation of serum biochemical marker concentrations and survival time in dogs with protein losing enteropathy. J Am Vet Med Assoc. 2015;246:91‐99. 25517330 10.2460/javma.246.1.91

[8] Gianella P , Lotti U , Bellino C , et al. Clinicopathologic and prognostic factors in short‐ and long‐term surviving dogs with protein‐losing enteropathy. Schweiz Arch Tierheilkd. 2017;159:163‐169. 28248185 10.17236/sat00108

[9] Allenspach K , Rizzo J , Jergens A , et al. Hypovitaminosis D is associated with negative outcome in dogs with protein losing enteropathy: a retrospective study of 43 cases. BMC Vet Res. 2017;13:96. 28390394 10.1186/s12917-017-1022-7 PMC5385077

[10] Rudinsky A , Howard J , Bishop M , et al. Dietary management of presumptive protein‐losing enteropathy in Yorkshire terriers. J Small Anim Pract. 2017;58:103‐108. 28160309 10.1111/jsap.12625

[11] Day MJ , Bilzer T , Mansell J . Histopathological standards for the diagnosis of gastrointestinal inflammation in endoscopic biopsy samples from the dog and cat: a report from the World Small Animal Veterinary Association Gastrointestinal Standardization Group. J Comp Pathol. 2008;138:S1‐S43. 18336828 10.1016/j.jcpa.2008.01.001

[12] Liverani E , Scaioli E , Digby RJ , Bellanova M , Belluzzi A . How to predict clinical relapse in inflammatory bowel disease patients. World J Gastroenterol. 2016;22(3):1017‐1033. 26811644 10.3748/wjg.v22.i3.1017 PMC4716017

[13] Mandigers PJ , Biourge V , van Den Ingh TS , et al. A randomized, open‐label, positively controlled field trial of a hydrolyzed protein diet in dogs with chronic small bowel enteropathy. J Vet Intern Med. 2010;24:1350‐1357. 21054541 10.1111/j.1939-1676.2010.0632.x

[14] Marchesi MC , Timpano CC , Busechian S , Pieramati C , Rueca F . The role of diet in managing inflammatory bowel disease affected dogs: a retrospective cohort study on 76 cases. Vet Ital. 2017;53:297‐302. 29307123 10.12834/VetIt.566.2700.1

[15] Wennogle SA , Stockman J , Webb CB . Prospective evaluation of a change in dietary therapy in dogs with steroid‐resistant protein‐losing enteropathy. J Small Anim Pract. 2021;62:756‐764. 33851420 10.1111/jsap.13334

[16] Riley SA , Mani V , Goodman MJ , Dutt S , Herd ME . Microscopic activity in ulcerative colitis: what does it mean? Gut. 1991 Feb;32(2):174‐178. 1864537 10.1136/gut.32.2.174 PMC1378803

[17] Burgener IA , Konig A , Allenspach K , et al. Upregulation of toll‐like receptors in chronic enteropathies in dogs. J Vet Intern Med. 2008;22:553‐560. 18466244 10.1111/j.1939-1676.2008.0093.x

[18] Okanishi H , Yoshioka R , Kagawa Y , Watari T . The clinical efficacy of dietary fat restriction in treatment of dogs with intestinal lymphangiectasia. J Vet Intern Med. 2014;28(3):809‐817. 24673630 10.1111/jvim.12327 PMC4238835

[19] Nagata N , Ohta H , Yokoyama N , et al. Clinical characteristics of dogs with food‐responsive protein‐losing enteropathy. J Vet Intern Med. 2020;34(2):659‐668. 32060974 10.1111/jvim.15720 PMC7096654

[20] Hall EJ , German AJ . Diseases of the small intestine. In: Ettinger SJ , Feldman EC , eds. Textbook of Veterinary Internal Medicine. 7th ed. Philadelphia, PA: WB Saunders; 2009:1333‐1378.

[21] Elkholly DA , Brodbelt DC , Church DB , et al. Side effects to systemic glucocorticoid therapy in dogs under primary veterinary care in the UK. Front Vet Sci. 2020;7:515. 32923470 10.3389/fvets.2020.00515 PMC7457010

[22] Kathrani A , Sanchez‐Vizcaino F , Hall EJ . Association of chronic enteropathy activity index, blood urea concentration, and risk of death in dogs with protein‐losing enteropathy. J Vet Intern Med. 2019;33:536‐543. 30784115 10.1111/jvim.15448 PMC6430906

[23] Gow AG , Else R , Evans H , Berry JL , Herrtage ME , Mellanby RJ . Hypovitaminosis D in dogs with inflammatory bowel disease and hypoalbuminaemia. J Small Anim Pract. 2011;52:411‐418. 21797872 10.1111/j.1748-5827.2011.01082.x

